# The effect of intrapartum antibiotics on early-onset neonatal sepsis in Dhaka, Bangladesh: a propensity score matched analysis

**DOI:** 10.1186/1471-2431-14-104

**Published:** 2014-04-17

**Authors:** Grace J Chan, Elizabeth A Stuart, Marzia Zaman, Abdullah A Mahmud, Abdullah H Baqui, Robert E Black

**Affiliations:** 1 Department of Medicine, Boston Children’s Hospital, Boston, USA; 2 Department of Global Health and Population, Harvard School of Public Health, Boston, USA; 3 Department of Mental Health, Johns Hopkins Bloomberg School of Public Health, Baltimore, USA; 4 Public Health Sciences Division, International Center for Diarrheal Disease Research, Dhaka, Bangladesh; 5 Department of International Health, Johns Hopkins Bloomberg School of Public Health, Baltimore, USA

**Keywords:** Intrapartum antibiotics, Early-onset neonatal sepsis, Propensity scores, Bangladesh

## Abstract

**Background:**

We estimate the effect of antibiotics given in the intrapartum period on early-onset neonatal sepsis in Dhaka, Bangladesh using propensity score techniques.

**Methods:**

We followed 600 mother-newborn pairs as part of a cohort study at a maternity center in Dhaka. Some pregnant women received one dose of intravenous antibiotics during labor based on clinician discretion. Newborns were followed over the first seven days of life for early-onset neonatal sepsis defined by a modified version of the World Health Organization Young Infants Integrated Management of Childhood Illnesses criteria.

Using propensity scores we matched women who received antibiotics with similar women who did not. A final logistic regression model predicting sepsis was run in the matched sample controlling for additional potential confounders.

**Results:**

Of the 600 mother-newborn pairs, 48 mothers (8.0%) received antibiotics during the intrapartum period. Seventy-seven newborns (12.8%) were classified with early-onset neonatal sepsis. Antibiotics appeared to be protective (odds ratio 0.381, 95% confidence interval 0.115–1.258), however this was not statistically significant. The results were similar after adjusting for prematurity, wealth status, and maternal colonization status (odds ratio 0.361, 95% confidence interval 0.106–1.225).

**Conclusions:**

Antibiotics administered during the intrapartum period may reduce the risk of early-onset neonatal sepsis in high neonatal mortality settings like Dhaka.

## Background

Neonatal infections - including sepsis, pneumonia, and meningitis - account for approximately 23.4% of the world’s 3.1 million neonatal deaths each year [1]. In developing countries, where 99% of neonatal deaths occur, up to 42% of infection related deaths occur in the first week of life [2]. This narrow time period provides only a small window of opportunity for interventions.

In Bangladesh, the incidence of clinical sepsis during the first week of life defined by the World Health Organization (WHO) Young Infants criteria for very severe disease [3] was 13.4% with a case-fatality of 10.2% [4] and the incidence of community-acquired neonatal bacteremia was 1.4 per 1000 live births [5]. The most common pathogen isolated was *S. aureus*[5].

Maternal infections and risk factors for infection or colonization increase the possibility of early-onset neonatal infections by vertical transmission [6]. Several interventions have been proposed to decrease the transmission of bacterial pathogens from the mother to newborn, particularly in preventing Group B Streptococcus (GBS) early-onset neonatal sepsis. For example, vaccines against the nine identified GBS stereotypes have been developed [7] and are currently being tested [8]. Another strategy is vaginal washes with chlorhexidine, which have been shown to reduce GBS bacterial load but did not affect early-onset sepsis [9,10]. The most commonly used intervention is antibiotics given during the intrapartum period.

Administration of antibiotics during the intrapartum period (most often penicillin) decreases vaginal GBS colony counts [11] and is thought to decrease the concentration of bacteria in the maternal bloodstream and amniotic fluid. Currently, in high income countries, indications for intrapartum antibiotic prophylaxis to prevent GBS early-onset neonatal sepsis are based on universal culture-based screening [12]. After implementation of the initial guidelines in 1996, a decreasing GBS incidence has been observed over time (1.7 per 1000 live births in 1993 compared to 0.34–0.37 per 1000 in recent years) [12-14].

Although the use of antibiotics during the intrapartum period has been widely adopted in high income countries, the evidence supporting antibiotic use derives mainly from cohort studies. There are limited data from randomized controlled trials and most are on GBS. A recent Cochrane review on antibiotics during the intrapartum period for known GBS maternal colonization identified only four randomized controlled trials, most of which were from the 1980s to early 2000s. Intrapartum antibiotics appeared to reduce GBS early-onset sepsis, however these findings may have been the result of a high risk of bias in the studies. The review concluded there was insufficient evidence to recommend intrapartum antibiotics to reduce GBS early-onset neonatal sepsis [15]. Furthermore, in regions like South Asia where the incidence of GBS early-onset sepsis was 0.02 per 1000 live births [16], it is unclear whether intrapartum antibiotics would reduce sepsis from other organisms.

Because there is a lack of randomized controlled trials and a dearth of data in particular from developing countries, we estimate the causal effect of intrapartum antibiotics on early-onset neonatal sepsis using propensity score matching in a cohort study of mother-newborn pairs in Dhaka, Bangladesh. Understanding the effect of intrapartum antibiotics on early-onset neonatal sepsis may lead to strategies to prevent sepsis and its associated morbidities and mortality globally.

## Methods

### Ethics statement

This study received ethical approval from the Johns Hopkins Bloomberg School of Public Health Committee on Human Research and the International Center for Diarrheal Disease Research, Bangladesh Ethical Review Committee. All study participants provided written informed consent. Parents or guardians gave informed consent on behalf of their newborns. Pregnant women in active labor initially provided verbal consent and then full written consent after delivery.

This study analyzes data collected as part of a cohort study, Maternal Origins of Neonatal Infection (MONI), which followed 600 mother-newborn pairs from January 15, 2011 to October 31, 2011 at a maternity center operated by Shimantik, a partner non-governmental organization in Dhaka, Bangladesh.

In the cohort study, pregnant women who planned to deliver at the maternity center were enrolled after 30 weeks gestation. Women with fetal distress, obstructed labor, hemorrhage, or severe pre-eclampsia were excluded to facilitate their need for urgent care. Women with antibiotic or steroid use two weeks before labor were excluded. Newborns who were delivered by caesarean section were excluded since the route of bacterial transmission differs by type of delivery and all women who delivered by caesarean sections at this facility received antibiotics. Newborns with birth injuries or surgical conditions requiring urgent care were excluded. Newborns were followed over the first seven days of life.

As part of the cohort study, Shimantik recruited four paramedics and five community health workers for primary data collection. Paramedics completed higher secondary school (12 years) and the national paramedics course. Community health workers finished at least secondary school (10 years). Two medical officers, one for field supervision and the other for quality assurance, were part of the study team. Staff received a two-week intensive training course by a pediatrician and local medical officer using the WHO Caring for the Newborn at Home training course for community health workers [17]. Sessions included presentations on basic principles, exercises and role plays on the recognition of clinical signs and symptoms, and field experiences in homes. Written exams and standardized observations were initially conducted and periodically repeated to maintain high levels of staff competency.

Demographic factors that may influence the receipt of antibiotics during labor and the incidence of early-onset sepsis were collected. Study paramedics collected data on maternal education, maternal age, antenatal care provider type, and receipt of tetanus toxoid as a proxy for access to health care. Wealth quintiles were created using principal components analysis with the following variables: construction of household materials, type of latrine, source of water, household number, number of children under five living in the household, and number of rooms where household members sleep [18].

At least one study paramedic was present around the clock in the labor and delivery room to assess maternal risk factors: stage and duration of labor, rupture of membranes, intrapartum temperature, number of vaginal exams performed, amniotic fluid color, hand washing by health workers, and maternal reproductive tract colonization status during labor. Women with a positive bacterial vaginal culture or positive GBS rectal culture were classified as colonized. Culture results were not available prior to delivery. Paramedics also collected data on neonatal characteristics such as sex, birth weight to the nearest 100 grams, and gestational age based on ultrasound report or maternal report of the date of last menstrual period.

During labor, some women were given one dose of intravenous antibiotics based on clinician discretion. Possible indications for antibiotics included episiotomy, failed trial of labor at home or delivery center, or early rupture of membranes. In Bangladesh, there are currently no established protocols for intrapartum antibiotic administration. Study paramedics observed and recorded any maternal receipt of intrapartum antibiotics.

The primary outcome measure was early-onset neonatal sepsis defined as a positive blood culture or classification of very severe disease by a physician or a community health worker following a modified version of the WHO Young Infants Integrated Management of Childhood Illnesses criteria (not able to feed or suck, history of convulsions, movement only when stimulated, respiratory rate >60 per min, severe chest indrawing, axillary temperature ≥37.5°C, axillary temperature ≤35.5°C) [3] without the diagnosis of asphyxia. Newborns were examined by a study physician before discharge from the maternity center and at home during days of life three and seven by community health workers. On days of life two, four, five, and six, community health workers conducted phone follow-ups. Newborns identified as sick by community health workers were evaluated by a study physician.

### Statistical analysis

We used propensity score matching to create groups of antibiotic-treated pregnant women and control (not receiving antibiotics) pregnant women who were similar with respect to observed characteristics [19]. Propensity scores, which reflect each pregnant woman’s predicted probability of receiving intrapartum antibiotics given a set of observed covariates, were created by a logistic regression model predicting receipt of antibiotics as a function of baseline characteristics and maternal risk factors. See Table 1 for a list of the 23 covariates used to create the propensity scores.

**Table 1:** Covariates by treatment (intrapartum antibiotics) and outcome (neonatal sepsis)

	**Treatment - intrapartum antibiotics**					**Outcome - sepsis**				
	**# no abx (n)**	**%**	**# with abx (n)**	**%**	**p-value**	**# no sepsis (n)**	**%**	**# with sepsis (n)**	**%**	**p-value**
Total	552		48			523		77		
Preterm	62	11.23	4	8.33		53	10.13	13	16.88	
No preterm	398	72.1	38	79.17	0.468	386	73.8	50	64.94	0.060
Missing	92	16.67	6	12.5	0.574	84	16.06	14	18.18	0.158
Low birth weight <2500 grams	92	16.67	7	14.58		85	16.25	14	18.18	
Not low birth weight	419	75.91	38	79.17	0.681	398	76.1	59	76.62	0.742
Missing	41	7.43	3	6.25	0.878	40	7.65	4	5.19	0.703
No tetanus toxoid	58	10.51	4	8.33		52	9.94	10	12.99	
Received tetanus toxoid	493	89.31	43	89.58	0.663	467	89.67	67	87.01	0.419
Missing	1	0.18	1	2.08	0.082	2	0.38	0	0	0.622
Antenatal care from a provider other than doctor	409	74.09	25	52.08		376	71.89	58	75.32	
Antenatal care from doctor	123	22.28	21	43.75	0.001	127	24.28	17	22.08	0.630
Missing	20	3.62	2	4.17	0.003	20	3.82	2	2.6	0.771
Mom no schooling	114	20.65	6	12.5		100	19.12	20	25.97	
Mom schooling	438	79.35	41	85.42	0.195	422	80.96	57	74.03	0.163
Missing	0	0	1	2.08	0.001	1	0.19	0	74.03	0.351
Maternal age < =22	272	49.28	22	45.83		253	48.37	41	53.25	
Maternal age >22	280	50.72	26	54.17	0.647	270	51.63	36	46.75	0.425
Roof tin, straw, leaf, bamboo	431	78.08	32	66.67		399	76.29	64	83.12	
Roof concrete, brick, cement	121	21.92	16	33.33	0.071	124	23.71	13	16.88	0.183
Wall tin, straw, leaf, bamboo, mud	216	39.13	14	29.17		199	38.05	31	40.26	
Wall concrete, brick, cement	336	60.87	34	70.83	0.173	324	61.95	46	59.74	0.710
Floor semi concrete, wood, straw, leaf, bamboo, mud	117	21.2	7	14.58		110	21.03	14	18.18	
Floor concrete	435	78.8	41	85.42	0.278	413	78.97	63	81.82	0.564
Household number >3	281	50.91	19	39.58		255	48.76	45	58.44	
Household number < =3	271	49.09	28	58.33	0.168	267	51.05	32	41.56	0.116
Missing	0	0	1	2.08	0.001	1	0.19	0	0	0.270
Number of children under 5 > 0	131	23.73	13	27.08		122	23.33	22	28.57	
Number of children under 5 = 0	421	76.27	34	70.83	0.545	400	76.48	55	71.43	0.319
Missing	0	0	1	2.08	0.003	1	0.19	0	0	0.565
Household latrine slab or hanging	210	38.04	13	27.08		192	36.71	31	40.26	
Household latrine sanitary	342	61.96	34	70.83	0.157	330	63.1	46	59.74	0.556
Missing	0	0	1	2.08	0.001	1	0.19	0	0	0.781
Household drinking water source tube	249	45.11	15	31.25		223	42.64	41	53.25	
Household drinking water source tap	303	54.89	32	66.67	0.080	299	57.17	36	46.75	0.082
Missing	0	0	1	2.08	0.001	1	0.19	0	0	0.205
Wealth (upper quintile of wealth)*	118	21.38	16	33.33		121	23.14	13	16.88	
Wealth (lower four quintiles)	434	78.62	31	64.58	0.045	401	76.67	64	83.12	0.431
Missing	0	0	1	2.08	<0.001	1	0.19	0	0	0.216
Active labor	239	43.3	15	31.25		216	41.3	38	49.35	
Early labor	274	49.64	29	60.42	0.110	269	51.43	34	44.16	0.190
Missing	39	7.07	4	8.33	0.269	38	7.27	5	6.49	0.409
Time in labor > =8 hours	277	50.18	22	45.83		257	49.14	42	54.55	
Time in labor < 8 hours	263	47.64	26	54.17	0.468	254	48.57	35	45.45	0.487
Missing	12	2.17	0	0	0.449	12	2.29	0	0	0.317
Rupture of membranes at presentation	215	38.95	29	60.42		216	41.3	28	36.36	
No rupture of membranes at presentation	326	59.06	19	39.57	0.005	296	56.6	49	63.64	0.333
Missing	11	1.99	0	0	0.012	11	2.1	0	0	0.273
Premature rupture of membranes	50	9.06	6	12.5		47	8.99	9	11.69	
No premature rupture of membranes	491	88.95	42	87.5	0.461	465	88.91	68	88.31	0.484
Missing	11	1.99	0	0	0.466	11	2.1	0	0	0.342
Amniotic Fluid green or cloudy	97	17.57	6	12.5		91	17.4	12	15.58	
Amniotic Fluid clear	432	78.26	40	83.33	0.369	409	78.2	63	81.82	0.643
Missing	23	4.17	2	4.17	0.668	23	4.4	2	2.6	0.683
Maternal temperature > =99	27	4.89	1	2.08		24	4.59	4	5.19	
Maternal temperature <99	492	89.13	47	97.92	0.340	470	89.87	69	89.61	0.819
Missing	33	5.98	0	0	0.136	29	5.54	4	5.19	0.966
Number of vaginal exams performed > =3	263	47.64	27	56.25		251	47.99	39	50.65	
Number of vaginal exams < 3	276	50	20	41.67	0.255	529	49.52	37	48.05	0.733
Missing	13	2.36	1	2.08	0.519	13	2.49	1	1.3	0.766
No hand washing before vaginal exam	134	24.28	15	31.25		130	24.86	19	24.68	
Hand washing before exam	395	71.56	32	66.67	0.323	370	70.75	57	74.03	0.853
Missing	23	4.17	1	2.08	0.474	23	4.4	1	1.3	0.424
No hand washing before delivery	34	6.16	2	4.17		31	5.93	5	6.49	
Hand washing before delivery	497	90.04	44	91.67	0.580	470	89.87	71	92.21	0.895
Missing	21	3.8	2	4.17	0.852	22	4.21	1	1.3	0.459
Colonization	202	36.59	17	35.42		184	35.18	35	45.45	
No colonization	337	61.05	29	60.42	0.944	326	62.33	40	51.95	0.080
Missing	13	2.36	2	4.17	0.741	13	2.49	2	2.6	0.209
Antibiotics*						45	8.6	3	3.9	
No antibiotics						478	91.4	74	96.1	0.155

*Not used to calculate propensity score.

We initially considered three matching methods: one-to-one matching with replacement, full constrained matching, and full unconstrained matching and selected the one method that best balanced covariates between the treated and control group. One-to-one matching selected for each treated woman the control woman with the most similar propensity score. After each match, the selected control was replaced into the control group and available for subsequent matching (i.e., matching was done “with replacement”). Controls not selected as a match were discarded and not used in subsequent analyses. Full matching retains all individuals and creates subgroups with at least one treated and one control with similar propensity scores. Treated and control individuals who did not have a good match (were outside the range of the propensity scores of the other group) were discarded. Following full matching, a weighting approach was used to account for multiple treated and control individuals in each subgroup, as described below. Full unconstrained matching did not limit the number of treated and control individuals in each subgroup while constrained full matching restricted the maximum number of controls to 10 per treated [20-22].

To check balance, we calculated the standardized difference for each covariate: the difference in means between the treated and control groups divided by the standard deviation in the control group, calculated before and after matching. We choose the matching method that yielded the smallest standardized bias across most of the covariates before running the final outcome regression models.

These methods estimated the average treatment effect among the treated individuals, in other words, the average outcome among the treated individuals compared to if they had not been treated [23]. In full matching, where there may be multiple treated and controls within each matched set, we utilized a weighted approach where control individuals are weighted to the treatment group. In particular, within each matched set, treated individuals received a weight of one. Control individuals received a weight proportional to the number of treated individuals in their matched set divided by the number of control individuals in their matched set, with the control weights scaled to sum to the total number of controls matched in the data [24]. These weights were then used in the final logistic regression model estimating the effect of intrapartum antibiotics on early-onset neonatal sepsis.

For variables with missing data, we imputed the mean of the variable. For those variables with more than 5% missing values we included in the propensity score model the variable itself as well as a missing data indicator [25]. We conducted a sensitivity analysis comparing the estimates resulting from this approach with those from a complete case analysis that excluded observations with missing data.

We also present results with a traditional logistic regression models without propensity score matching for comparison. In both models, the propensity score matched and traditional logistic regression without propensity score matching, we performed a crude analysis as well as an adjusted analysis controlling for the potential confounders that were associated with sepsis (p < 0.10): prematurity, maternal colonization status, and wealth status.

The dataset was prepared using STATA v12 (StataCorp, College Station, TX). Statistical analyses were conducted in R, version 2.14.0, with the propensity score matching conducted using the MatchIt package [24].

## Results

Of the 600 mother-newborn pairs enrolled, 48 mothers (8.0%) received intrapartum antibiotics. The most commonly used intravenous antibiotics were one dose of cephalexin 500 mg (79.1%), amoxicillin 500 mg (16.7%), or penicillin 500 mg (4.2%). Seventy-seven newborns (12.8%) were classified with early-onset neonatal sepsis; three of whom were born to treated mothers. Physicians diagnosed or confirmed the diagnosis in 44 newborns. Kappa statistics show substantial agreement (к = 0.63) between assessments of very severe disease by community health workers and physicians. All peripheral blood cultures (n = 12) obtained among newborns diagnosed with clinical early-onset neonatal sepsis were negative. The most common organisms detected from maternal vaginal cultures were *S. aureus* (7.4%), Non-GBS streptococcus (6.8%), and GBS (6.2%).

Several baseline characteristics were associated with receipt of intrapartum antibiotics and early-onset neonatal sepsis (Table 1). Factors associated with intrapartum antibiotic use included receipt of antenatal care from physicians (43.8% vs. 22.3%, p = 0.001), homes with roofs made of concrete, brick, or cement (33.3% vs. 21.9%, p = 0.07), drinking water sources from the tap rather than tube well (66.7% vs. 54.9%, p = 0.08), upper quintile of wealth (33.3% vs. 21.4%, p = 0.05), and rupture of membranes at presentation (60.4% vs. 39.0%, p = 0.005). Characteristics associated with early-onset sepsis were prematurity (16.9% vs. 10.1%, p = 0.06), colonized mothers (45.5% vs. 35.2%, p = 0.08), and homes with drinking water sources from a tube well rather than tap (53.3% vs. 42.6%, p = 0.08).

Across the three matching methods considered, full unconstrained matching had the best overall balance across the covariates. After matching, the absolute standardized biases ranged from −0.19 to 0.18. The variable with the maximum standardized difference (−0.19) was no hand washing before vaginal exam. See Additional file 1: Table S1 for a summary of balance for matched and unmatched data. The full unconstrained method matched 500 controls and 48 treated women (52 controls were discarded).

Using the propensity score matched dataset (n = 548), there was a reduction in sepsis rates, although not statistically significant, between newborns of mothers who received intrapartum antibiotics and newborns of mothers who did not receive intrapartum antibiotics (odds ratio [OR] 0.381, 95% confidence interval [CI] 0.115–1.258). The result was similar after adjusting for prematurity, wealth status, and maternal colonization status (OR 0.361, 95% CI 0.106–1.225) (Table 2).

**Table 2:** Effect of intrapartum antibiotics and early-onset neonatal sepsis models: propensity score (PS) matched adjustment, propensity score matched adjustment complete case analysis, and traditional logistic regression no propensity score matching

**Model**	**Treated**	**Controls**	**OR**	**95% CI**
Propensity score matched unadjusted	48	500	0.381	0.115–1.258
Propensity score matched adjusted*	48	500	0.361	0.106–1.225
PS matched complete case analysis unadjusted	38	280	0.160	0.021–1.197
PS matched complete case analysis adjusted*	38	280	0.170	0.022–1.295
Traditional logistic regression no PS matching unadjusted	48	552	0.431	0.130–1.421
Traditional logistic regression no PS matching adjusted*	41	451	0.458	0.138–1.521

*The adjusted analyses controlled for prematurity, wealth status, and maternal colonization status.

We conducted a sensitivity analysis with a complete case dataset (n = 408) that excluded observations with missing data. Matching with the full unconstrained method yielded 280 controls and 38 treated women (90 controls were discarded). Again there was a reduction, not statistically significant, in sepsis rates between the antibiotic group compared to the control group (OR 0.160, 95% CI 0.021–1.197). The results were similar after adjusting for prematurity, the highest wealth quintile, and maternal colonization status (OR 0.170, 95% CI 0.022–1.295).

Analysis with traditional logistic regression models (n = 600) without propensity score matching showed similar results. There was a reduction in sepsis rates, not statistically significant, between the antibiotic and control groups (OR 0.431, 95% CI 0.130–1.421), with similar results after adjusting for prematurity, the highest wealth quintile, and maternal colonization status (OR 0.458, 95% CI 0.138–1.521).

Since the number of sepsis cases in the treated group were small, we also compared p-values from a Fisher’s exact test of treatment and sepsis (p = 0.182) with the propensity score unmatched logistic regression (p = 0.167) and found little difference.

## Discussion

Antibiotics during labor suggest a decreased risk, although not statistically significant, of early-onset neonatal sepsis in this population. A reduction of early-onset neonatal sepsis by 64%, if confirmed, is clinically important.

Our findings are robust across the different approaches and methods with similar point estimates and confidence intervals. The propensity score matched adjustment estimate is somewhat larger in magnitude compared to the result from traditional regression analysis. Prior to propensity score matching, the observed covariates were imbalanced between the treated and control groups, particularly rupture of membranes at presentation and antenatal care provider type. Propensity score matching reduced confounding by indication by achieving better balance of the observed covariates across the treated and control groups. We further adjusted for confounders by fitting a regression model assuming a normal logistic regression of sepsis given antibiotic use and the observed covariates.

Our sensitivity analysis, a complete case analysis rather than a single imputation of missing values, further decreased the number of sepsis cases in the treatment group (to 1) which may have contributed to a more protective odds in that sensitivity analysis suggesting that our data were missing not at random.

There are few randomized controlled trials that examined intrapartum antibiotics and early-onset neonatal sepsis. A study by Matorras et al. (1990) in Spain found that administration of intrapartum ampicillin to GBS colonized women decreased GBS positive culture cases of early-onset sepsis by 85% and cases of clinical early-onset sepsis by 78% [26]. In Finland, a study by Tuppurainen (1989) found that administering penicillin to GBS colonized women during labor reduced GBS positive culture cases of early-onset sepsis by 75% and cases of clinical early-onset sepsis by 83% [27]. However, in neither study was the reduction statistically significant (a similar scenario as what we found here). One randomized controlled trial by Gibbs (1988) found that ampicillin and gentamicin administered to women with intra-amniotic infections during labor was associated with a statistically significant reduction in early-onset neonatal sepsis (clinical or culture confirmed) (OR 0.04, 95% CI 0.00–0.75) [28]. All three of these studies targeted high risk pregnant women with intra-amniotic infections or colonization with GBS.

In our study, we included all women regardless of their risk status. In low-resource settings like Bangladesh the ability to implement a universal screen using vaginal swabs would be limited. However, a risk factor and clinical symptoms screen may be feasible and useful to test in such a setting.

Antibiotic choice depends on local knowledge of the etiology of maternal colonization and neonatal sepsis and antibiotic resistance patterns. In our facility, most women received the standard first generation cephalosporin, cephalexin, which provides excellent gram positive coverage. An earlier study at the same facility found that the most prevalent organism in the maternal vaginal tract was *S. aureus*, which was also one of the most common etiologies of neonatal bacteremia in Bangladesh [5]. However, approximately 25% of *S. aureus* isolates were resistant to cephalexin in a community-based study in Bangladesh [5].

The study has several limitations. We assume there are no unobserved differences between the treatment and control groups given the observed variables; we are only able to adjust for observed confounders. We had more than 5% missing data on gestational age, birth weight, and maternal temperature; we used sensitivity analysis to examine robustness across how we handled this missing data. We had limited neonatal blood culture data due to low compliance with referrals to tertiary care centers. Therefore, our primary outcome measure of neonatal sepsis relied on clinical signs and symptoms, which is overly sensitive and nonspecific. However, newborns of mothers with or without intrapartum antibiotics were non-differentially classified as having sepsis. This non-differential misclassification would have underestimated our effect size. As stated above, our dataset is of relatively small size. Our effective sample size following matching was 89 controls and 48 treated, implying that we had less than 80% power to detect an effect of the size that we found. But the data provides our best information at this point about these associations. Additional, and larger, studies are needed to confirm these results. The provision of antibiotics was based on clinician discretion without a set protocol, which allowed us to mimic randomization of women to antibiotics or control based on a set of similar observed characteristics.

To our knowledge, this is the first study that uses propensity scores to determine the effects of intrapartum antibiotics on early-onset neonatal sepsis in developing country settings. Given the challenges of conducting a randomized controlled trial in settings where intrapartum antibiotic prophylaxis is widely accepted for GBS colonization like the United States and the lack of preliminary data in countries like Bangladesh, this is an attractive method that represents a strong design to investigate the causal effects of antibiotic prophylaxis and early-onset neonatal sepsis [19].

Including intrapartum antibiotics as part of a comprehensive neonatal survival package has the potential to save many lives. Given the potential for tremendous benefit, a double-blind randomized controlled trial testing the effect of intrapartum antibiotics on early-onset neonatal sepsis or larger studies using propensity score matching would be ideal. Current evidence from randomized controlled trials is inconclusive and limited only to GBS [29]. In designing future studies, we need to examine the frequency and timing of antibiotic doses required, the optimal antibiotic choice depending on the geographic variability of organisms, and possible risks. Future studies would provide needed knowledge for areas where intrapartum antibiotics prophylaxis is currently given and support or nullify the use of intrapartum antibiotics in low-resource settings.

## Conclusion

Antibiotics during labor indicated a strong protection against early-onset neonatal sepsis (OR = 0.36), but the relative size of our population did not yield a level of statistical significance. In settings where the burden of neonatal mortality is disproportionately high, additional studies with larger datasets using propensity scores or randomized controlled trials testing the effect of intrapartum antibiotics on early-onset neonatal sepsis are warranted.

## Competing interests

The authors declare that they have no competing interests.

## Authors’ contributions

Each author made substantial contributions to the study. GC designed the study and wrote the first draft of the manuscript. GC and ES conducted the statistical analysis. MZ and AM supervised the field team and acquisition of data. AB and RB provided technical expertise and interpretation of the data. All authors were involved in manuscript revisions and approved the final manuscript.

## Pre-publication history

The pre-publication history for this paper can be accessed here:

http://www.biomedcentral.com/1471-2431/14/104/prepub

## Supplementary Material

Additional file 1: Table S1 Summary of balance for unmatched and matched data with single imputation of missing data. Click here for file
