# Supplementary material for: The effect of intrapartum antibiotics on early-onset neonatal sepsis in Dhaka, Bangladesh: a propensity score matched analysis
Source: BMC Pediatr. 2014 Apr 17;14:104. doi: 10.1186/1471-2431-14-104 (PMC4021342; doi:10.1186/1471-2431-14-104)
Supplement: Additional file 1: Table S1 — Summary of balance for unmatched and matched data with single imputation of missing data. [file 1471-2431-14-104-S1.doc]

**Additional file 1: Table S1. Summary of balance for unmatched and matched data with single imputation of missing data**

|  | Unmatched data | | | Matched data | | |
| --- | --- | --- | --- | --- | --- | --- |
|  | Treated proportion | Control proportion | Standardized difference | Treated proportion | Control proportion | Standardized difference |
| Propensity score | 0.14 | 0.07 | 0.87 | 0.14 | 0.14 | 0.00 |
| Preterm | 0.10 | 0.13 | -0.12 | 0.10 | 0.10 | 0.01 |
| Low birth weight <2500 grams | 0.16 | 0.18 | -0.06 | 0.16 | 0.16 | 0.00 |
| No tetanus toxoid | 0.09 | 0.11 | -0.07 | 0.09 | 0.08 | 0.00 |
| Antenatal care from a provider other than doctor | 0.55 | 0.77 | -0.44 | 0.55 | 0.53 | 0.04 |
| Mom no schooling | 0.13 | 0.21 | -0.23 | 0.13 | 0.13 | -0.01 |
| Maternal age<=22 | 0.46 | 0.49 | -0.07 | 0.46 | 0.41 | 0.10 |
| Roof tin, straw, leaf, bamboo | 0.67 | 0.78 | -0.24 | 0.67 | 0.60 | 0.14 |
| Wall tin, straw, leaf, bamboo, mud | 0.29 | 0.39 | -0.22 | 0.29 | 0.28 | 0.03 |
| Floor semi concrete, wood, straw, leaf, bamboo, mud | 0.15 | 0.21 | -0.19 | 0.15 | 0.15 | 0.00 |
| Household number >3 | 0.41 | 0.51 | -0.21 | 0.41 | 0.39 | 0.04 |
| Number of children under 5 >0 | 0.28 | 0.24 | 0.09 | 0.28 | 0.25 | 0.06 |
| Household latrine slab or hanging | 0.28 | 0.38 | -0.23 | 0.28 | 0.27 | 0.03 |
| Household drinking water source tube | 0.32 | 0.45 | -0.28 | 0.32 | 0.34 | -0.05 |
| Active labor | 0.35 | 0.47 | -0.25 | 0.35 | 0.30 | 0.10 |
| Time in labor >= 8 hours | 0.46 | 0.51 | -0.11 | 0.46 | 0.42 | 0.08 |
| Rupture of membranes at presentation | 0.60 | 0.40 | 0.42 | 0.60 | 0.52 | 0.18 |
| Premature rupture of membranes | 0.13 | 0.09 | 0.10 | 0.13 | 0.11 | 0.05 |
| Amniotic Fluid green or cloudy | 0.13 | 0.18 | -0.15 | 0.13 | 0.12 | 0.04 |
| Maternal temperature >=99 | 0.02 | 0.05 | -0.22 | 0.02 | 0.02 | -0.02 |
| Number of vaginal exams performed >=3 | 0.57 | 0.49 | 0.17 | 0.57 | 0.56 | 0.03 |
| No hand washing before vaginal exam | 0.32 | 0.25 | 0.14 | 0.32 | 0.41 | -0.19 |
| No hand washing before delivery | 0.04 | 0.06 | -0.10 | 0.04 | 0.05 | -0.02 |
| Colonization | 0.37 | 0.37 | -0.01 | 0.37 | 0.36 | 0.02 |
| Preterm missing indicator | 0.13 | 0.17 | -0.12 | 0.13 | 0.13 | 0.00 |
| Low birth weight missing indicator | 0.06 | 0.07 | -0.05 | 0.06 | 0.05 | 0.04 |
| Active labor missing indicator | 0.08 | 0.07 | 0.05 | 0.08 | 0.09 | -0.02 |
| Maternal temperature missing indicator | 0.00 | 0.06 |  | 0.00 | 0.00 | 0.00 |
